# Cervical cancer in Morocco: a literature review on risk factors, prevalence, and healthcare challenges

**DOI:** 10.11604/pamj.2025.50.11.43975

**Published:** 2025-01-07

**Authors:** Malika Allali, Khaoula Errafii, Maryame Lamsisi, Karima Fichtali, Sanae El Majjaoui, Hicham El Fazazi, Najib Al Idrissi, Adil El Ghanmi, Lahcen Ghanmi, Bouchra Ghazi, Nabil Ismaili, Wajih Rhalem, Hassan Ghazal, Youssef Bakri, Salsabil Hamdi

**Affiliations:** 1 Virology and Public Health Laboratory, Serum and Vaccine Center (*Institut Pasteur du Maroc*), Casablanca, Morocco,; 2 Laboratory of Human Pathologies Biology, Department of Biology and Genomic Center of Human Pathologies, Faculty of Sciences, University Mohammed V, Rabat, Morocco,; 3 African Genome Center, Mohamed IV Polytechnic University, Benguerir 43151, Morocco,; 4 Biotechnology Unit, Serum and Vaccine Center (*Institut Pasteur du Maroc*), Casablanca, Morocco,; 5 Immunopathology-Immunotherapy-Immuno monitoring Laboratory, Faculty of Medicine, Mohammed VI University of Health Sciences (UM6SS), Casablanca, Morocco,; 6 Laboratory of Genomics, Genetics, Epigenetics, Precision and Predictive Medicines (PerMed), Faculty of Medicine, Mohammed VI University of Sciences and Health, Casablanca, Morocco,; 7 Fertility Center, Cheikh Zaid International University, Abulcasis International University of Health Sciences, Rabat, Morocco,; 8 Research Team E2SN, Ensam, Mohammed V University, Rabat, Morocco,; 9 Scientific Department, National Center for Scientific and Technical Research (CNRST), Rabat, Morocco

**Keywords:** Cervical cancer, diagnostic/screening, prevention, human papillomavirus, treatment, Morocco

## Abstract

Cervical cancer (CC) stands as the second most prevalent cancer among women in Morocco, with an estimated annual incidence of 2165 cases and over 1199 associated deaths. However, the lack of a comprehensive national registry requires caution in interpreting these figures. This review highlights the examination of the epidemiological profile, diagnostic modalities, risk factors, treatment, and prevention related to CC screening within the context of Morocco. High-risk subtypes of the human papillomavirus (HPV) are the etiological cause of the disease in most cases. Underscoring the urgent need for a thorough understanding of its epidemiological landscape. Other risk factors as age of sexual debut, sexual partners and sexually transmitted infections, immunosuppression, smoking, and parity should be studied comprehensively. Treatment modalities for CC are contingent upon disease staging at diagnosis and the availability of local healthcare resources. Options encompass radical hysterectomy and chemoradiation. The Ministry of Health's endorsement of the vaccine in 2022 marked a significant milestone in combating cancer through prevention and treatment, as it integrated the HPV vaccination into the national vaccination program, targeting girls aged 11 years. Morocco's healthcare landscape underwent significant reforms, culminating in the implementation of compulsory basic medical coverage in 2020 to ensure equitable access to healthcare services for all citizens. Consequently, this proactive approach holds promise for enhancing care standards and substantially curtailing mortality rates attributable to cancer across Morocco in the coming years.

## Introduction

Cervical cancer (CC) is the fourth most frequent female cancer, and it is one of the major global health issues in the world [1]. In 2015, low- and middle-income countries (LMICs) accounted for 90% of the 270,000 CC deaths, with mortality rates 18 times higher than in developed countries [2]. The disease's natural history is widely established. Most frequently asymptomatic and develops over several years. In fact, 40% to 50% of sexually active women are infected with Human Papillomavirus (HPV) [2]. Human Papillomavirus infection usually only lasts for eight months on average, clears after one year in 70% of instances, and after two years in 91% of cases. Precancerous lesions, which may regress or evolve into invasive CC, arise when an oncogenic HPV infection is persistent. It takes between 10 and 20 years for a precancerous lesion to develop into cancer. The most frequent histological subtypes of CC are squamous cell carcinoma and adenocarcinoma, which account for roughly 70% and 25% of all cases, respectively [3,4]. Human Papillomavirus screening and vaccination programs are effective preventative measures. HPV vaccination is the primary prevention of CC. There are three vaccines: Cervarix®, a bivalent vaccine, the quadrivalent vaccine Gardasil® [3,4], and the noncovalent Gardasil® 9. The HPV vaccination is able to prevent and potentially eliminate HPV-related malignancies such as CC. Secondary prevention of CC is based on the early detection and treatment of precancerous lesions [3,4].

Despite recent advances in CC prevention, screening, diagnosis, and treatment, the significant regional and global disparities in CC outcomes have prompted international gynecologic cancer societies to publish evidence-based management guidelines aimed at improving patient care quality [5]. WHO estimates to reduce the incidence of CC to 4 cases or less per 100,000 women per year by 2030 [6]. It plans several interventions including screening 70% of women aged 35 to 45, detecting cervical lesions in 90% of affected women to enable diagnosis and treatment, and vaccinating 90% of women aged 15 with the HPV vaccine [6]. Roughly 80,000 women are diagnosed with CC each year in Africa, where 267.9 million women aged 15 or older are at risk of developing the disease, the observed mortality rate is slightly over 60,000 cases per year [7]. The highest rates in Africa (ASIR > 40/<100,000) are seen in Eastern and Southern Africa. Furthermore, there are significant regional variations for Southern Africa [1], where the highest incidence is found in Lesotho and Swaziland, two countries with no screening programs or anticancer treatment facilities and only 1 and 2 doctors per 10,000 population, respectively (compared to 8/10,000 in South Africa and 27/10,000 in the United States) [8]. The morbidity of CC varies across countries in the Maghreb. It's important to note that these figures are based on available data and may not be strongly representative of the current situation. Indeed, Morocco had the greatest incidence of CC (16.3/100,000) [5], followed by Algeria (10.2/10,0000) [9]. One thousand three hundred and twenty-three (1323) new CC cases were reported in Algeria in 2015 [9].

The most recent publications in Tunisia's regional cancer registries relate to the years 2004-2006 for the northern region, 2003-2007 for the governorate of Sousse, and 2000-2002 for the governorate of Sfax; as a result, we used estimates from those registries. As the data from the regional cancer registries in Tunisia are not updated, we used estimates from GLOBOCAN 2012 [8]. In Morocco, only hospital registries are available. Casablanca, Rabat, and Fez are home to the country's major cancer centers. The Casablanca cancer facility opened in 1929, whereas Rabat's did so in 1985, and the Fez cancer center opened in 2005. According to records from Casablanca's Ibn Rochd Oncology Center, CC was nearly sole cancer among Moroccan women in the 1940s [10]. Until the 1990s, when a major increase in breast cancer in women was noticed, it remained the most frequent cancer in the population [10]. The National Cancer Institute of Rabat reported the same findings [10]. After breast cancer, CC is now the second most frequent cancer among women [11]. According to GLOBOCAN 2008 [12], the global age-standard incidence of CC in Moroccan women was 14.1 new cases per 100,000 people per year (1979 new cases per year). This cancer had an 8.4 per 100,000 mortality rate (1152). The most important independent prognostic factor is the stage of diagnosis [13,14]. Since its establishment in 2005, the Foundation Lalla Salma against Cancer has been working closely with its partners to address the pressing issue of cancer within Morocco and the broader region [15].

Among its initiatives, the foundation has conducted epidemiological studies on HPV and has actively supported the development of an extensive CC screening program. Various strategies are being implemented to prevent and control HPV infections, with a particular focus on promoting HPV immunization. In 2005, Morocco implemented a mandatory, contributory health insurance program known as AMO for formal sector workers. This initiative was augmented in 2012 with the introduction of a non-contributory basic coverage program called the Assistance Scheme Medical (RAMED) to cater to the informal sector. These schemes were formalized under Law 65.00 of the medical coverage code. In 2020, a significant reform occurred with the introduction of compulsory basic medical coverage, ensuring universal access to healthcare and promoting equity and fairness in healthcare access across the entire population [16]. The Moroccan government's steadfast efforts in healthcare have been instrumental in transforming the nation's healthcare landscape. This comprehensive approach not only ensures universal healthcare access but also fosters fairness and inclusivity in healthcare delivery, thereby enhancing the overall well-being of Morocco's populace. The value added by these government efforts lies in their profound impact on reducing disparities in healthcare access and promoting the fundamental right to health for all citizens [16]. Consequently, there will be an improvement in the care of patients and particularly CC in Morocco.

Cervical cancer is the second most prevalent cancer among women in Morocco, with its true prevalence likely underestimated due to the lack of a national registration system. The absence of screening programs contributes to delayed diagnosis, resulting in decreased treatment efficacy. However, the inclusion of the HPV vaccination into the national program, alongside enhanced health coverage, is expected to positively influence future treatment outcomes. Objectives; this review aims to evaluate CC screening within the context of Morocco by addressing the following questions: i) What is the epidemiological profile of CC in Morocco; ii) what diagnostic modalities are currently employed for CC screening in Morocco, and how effective are they; iii) what are the primary risk factors for CC among the Moroccan population; iv) how accessible and effective are the treatment options for CC in Morocco; v) what prevention strategies, such as vaccination and public health programs, have been implemented in Morocco, and what impact have they had?

## Methods

**Eligibility criteria:** this systematic literature review focused on CC screening within Morocco. Eligible studies included English-language, peer-reviewed articles and gray literature addressing the epidemiology, risk factors, diagnostic methods, treatment, and prevention strategies specific to the Moroccan context. Articles focusing exclusively on other cancers or unrelated to Morocco were excluded. No date restrictions were applied.

**Information sources:** the review utilized multiple information sources to ensure comprehensive coverage. Databases searched included PubMed, Web of Science, ScienceDirect, and Springer. Additional sources included Google Scholar for gray literature, such as PhD and master's theses and reports from organizations involved in CC research in Morocco. The initial search was conducted on 15 November 2020, with the final search performed on January 10, 2024.

**Search strategy:** the search strategy combined medical subject headings (MeSH) and keywords relevant to CC and Morocco. Search terms included “cervical cancer,” “Morocco,” “epidemiology,” “diagnosis,” “risk factors,” “treatment,” “prevention,” and “screening.” Boolean operators such as “AND” and “OR” were employed to refine the results. For example, the combination “Morocco” AND “cervical cancer” AND “epidemiology” produced targeted results, while “Morocco” OR “epidemiology” OR “diagnosis” broadened the search scope. The search strategy underwent iterative refinement based on feedback from epidemiologists and public health experts.

**Selection process:** two independent reviewers screened all retrieved records, including titles and abstracts, while blinded to journal titles, study authors, and institutions. Studies meeting inclusion criteria were retrieved as full-text articles for detailed evaluation. Discrepancies in the selection process were resolved through discussion, with a third reviewer consulted as needed.

**Data collection process:** data extraction was performed using a standardized form capturing key variables, including study location, design, outcomes, sample size, population characteristics, and primary findings. Both reviewers conducted the extraction independently, piloting the form to ensure consistency. Disagreements were resolved through consensus or consultation with additional reviewers.

**Data items:** the review collected data on various outcomes, including epidemiological trends, diagnostic practices, risk factors, treatment methods, and prevention strategies for CC in Morocco. Additional variables extracted from the studies included study design, population characteristics, sample size, and funding sources. When information was missing or unclear, assumptions were made based on available data, and where possible, efforts were made to contact study investigators to clarify uncertainties.

**Study risk of bias assessment:** the prediction model risk of bias assessment tool (PROBAST) was used to assess the risk of bias, chosen for its relevance to health prediction model studies. Both reviewers independently conducted bias assessments. Disagreements were resolved through consensus, with a third reviewer providing input when necessary.

**Figure 1:**
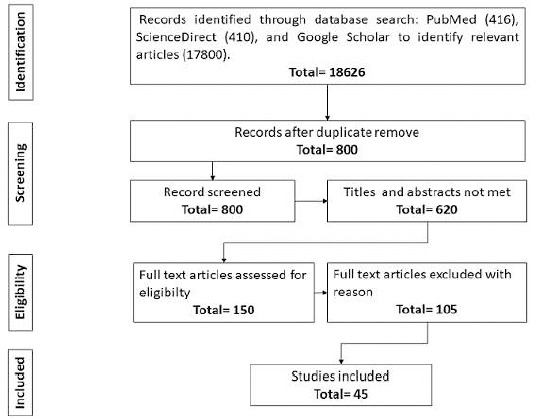
flow diagram of study selection process for literature review on cervical cancer in Morocco

**Synthesis methods:** due to heterogeneity in study settings, diagnostic methods, and reported outcomes, a meta-analysis was not performed. Instead, findings were synthesized narratively to summarize the epidemiological profile, diagnostic practices, risk factors, treatment strategies, and prevention initiatives for CC in Morocco. The narrative synthesis highlighted research gaps and public health opportunities (Figure 1).

## Results

**Study selection:** the study selection process is outlined in the PRISMA Flow Diagram (Figure 1). The initial search identified 9,000 records, of which 900 were randomly selected for screening. Following the removal of duplicates and irrelevant records, 890 studies were retained for full-text review based on the inclusion criteria. These studies addressed CC in Morocco, focusing on epidemiology, risk factors, prevention strategies, diagnostic methods, and treatment approaches. There were no unresolved disagreements regarding study inclusion or data extraction.

**Characteristics of included studies:** the characteristics of the included studies are summarized in Table 1. All studies used observational designs, including retrospective studies, cross-sectional analyses, and case-control studies. The studies were conducted across various Moroccan regions, including Rabat, Casablanca, Agadir, and Fez, with sample sizes ranging from 200 to over 9,000 cases. Most studies focused on the epidemiological profile of CC, reporting an annual incidence rate of 10.4 cases per 100,000 women and a mortality rate of 5.8 per 100,000. A significant subset of studies also explored the distribution of histological differentiation, with squamous cell carcinoma being the most common subtype (Table 2). Furthermore, data on the stages of CC at diagnosis revealed that 85.3% of cases were detected at intermediate or advanced stages, with only 14.7% identified at early stages. This highlights the challenges of late-stage diagnosis due to underscreening and limited early detection initiatives. The studies also examined risk factors, prevention strategies, and the impact of HPV infections. Multiple reports detailed associations between CC and high-risk HPV types, especially among HIV-positive women, with notable differences in prevalence rates based on demographic and clinical factors. Data collection methods and variables varied across studies, but all contributed to a comprehensive understanding of CC in Morocco.

**Table 1:** key epidemiological metrics of cervical cancer and HPV prevalence

Metric	Value
Annual Incidence of CC	10.4 cases per 100,000 women
Annual mortality rate of CC	5.8 per 100,000 women
Age-standardized incidence (2008)	14.1 per 100,000 women
Contribution to total cancer prevalence	17.2%
Mortality contribution	12.6%
HPV prevalence range in normal cytology	4.2%-42.5%

HPV: human papillomavirus; CC: cervical cancer

**Table 2:** distribution of histological differentiation in cervical cancer cases

Histological differentiation	Percentage
Well-differentiated	29.5%
Moderately differentiated	51.4%
Poorly differentiated	17.6%
Undifferentiated	1.5%

**Epidemiological profile of cervical cancer in Morocco:** the characterization of CC in Morocco is hindered by the absence of a national cancer registry, limited epidemiological studies, and the lack of a structured screening program. Available data indicate that CC is the second most prevalent cancer among women in Morocco, with an annual incidence rate of 10.4 cases per 100,000 and a mortality rate of 5.8 per 100,000 [17]. A summary of prevalence and incidence data is presented in Table 1.

**Key epidemiological metrics of cervical cancer and HPV prevalence:** a retrospective study conducted at INO in Rabat and the Oncology Department at Ibn Rochd Hospital in Casablanca reviewed 900 randomly selected cases out of 9,000 records from 2003-2007. Among these, 890 records were retained for analysis. Histological differentiation of CC cases showed the following distribution (Table 2) [18].

**Figure 2:**
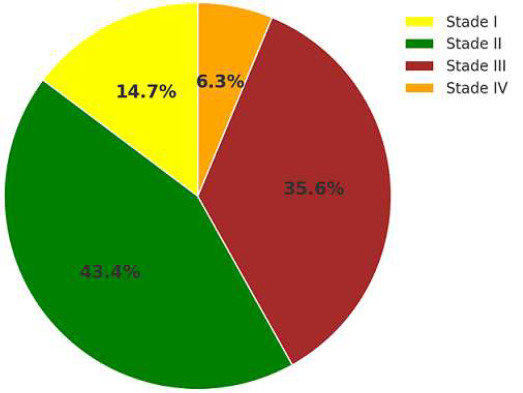
the average of two research “FIGO” classifications in Morocco

**Distribution of histological differentiation in cervical cancer cases:** the distribution of CC stages was reported in two studies conducted at INO in Rabat and Ibn Rochd Hospital. Among patients, 14.7% were diagnosed at early stages (IB2), while 85.3% were classified at intermediate or advanced stages: 43.25% in stage II, 35.6% in stage III, and 5.25% in stage IV. These distributions are typically represented in a pie chart format [18,19]. Additionally, studies conducted in Agadir and Casablanca examined the association between CC and Human Immunodeficiency Virus (HIV), reporting a higher prevalence of high-risk HPV infections among HIV-positive women compared to their HIV-negative counterparts [20,21]. The average of two research “FIGO” classifications in Morocco [22,23] (Figure 2).

**Risk factors for cervical cancer:** chronic infection by high-risk oncogenic subtypes of HPV is established as the origin of CC [22]. Human papillomavirus infection is a necessary but insufficient cause of CC, with persistent infection caused by high-risk HPV, combined with other cofactors, contributing to the pathogenesis. These cofactors include early age of sexual debut, multiple sexual partners, immunosuppression, history of sexually transmitted infections (STIs), smoking, parity, oral contraception, and non-attendance for screening [24,25].

**Age of sexual debut:** early initiation of sexual activity is a recognized risk factor for CC, as demonstrated by multiple studies in Morocco. A multicenter case-control study conducted between 2009 and 2012 reported that initiating sexual intercourse before the age of 18 was associated with a 2.4-fold increase in the risk of developing CC compared to those who began after 18 [25]. Similarly, research conducted in Rabat, involving 214 cases and 203 controls, identified sexual activity before the age of 15 as a statistically significant factor in increasing CC risk [26]. Furthermore, a 2011 study revealed that approximately 85% of participants reported their first sexual experience occurred between the ages of 14 and 19 [24,27]. These findings highlight the critical role of early sexual activity in influencing the risk of CC in the Moroccan population.

**Sexual partners:** the risk of CC is known to increase with a higher number of lifetime sexual partners, although findings in Morocco show some variability. A study conducted between 2009 and 2012 reported a 2.1-fold increased risk of CC among women who had two or more lifetime sexual partners [28]. However, contrasting results were observed in another study conducted in Rabat and Casablanca, which found no significant association between the number of sexual partners and the risk of CC [26]. These differing outcomes highlight the need for further research to better understand the relationship between sexual behavior and CC in the Moroccan context.

**Sexually transmitted infections:** co-infections with HPV and other sexually transmitted infections (STIs) are significant contributors to the risk of CC. In a study involving 438 Moroccan women, HPV infection was detected in 32.3% of participants, Chlamydia trachomatis in 17.7%, and co-infection in 13.4%. Co-infection was notably associated with an odds ratio (OR) of 3.18 for cervical abnormalities [29]. Additionally, HIV infection was found to markedly increase susceptibility to high-risk HPV types and abnormal Pap smear results among HIV-positive Moroccan women [21]. These findings emphasize the compounded risk posed by co-infections in the development of CC.

**Immunosuppression:** significantly heightens the risk of CC, as evidenced by studies conducted in Morocco. Research on HIV-positive women demonstrated a high prevalence of HPV infection, even among those with CD4 T-cell counts exceeding 200/mm^3^ [21]. This underscores the increased vulnerability of immunosuppressed individuals to HPV-related cervical abnormalities, highlighting the need for targeted interventions in this population.

**Smoking:** the role of smoking as a risk factor for CC in Morocco has produced mixed findings. A study conducted between 2009 and 2012 reported that smoking more than one packet per year was associated with an increased risk of CC, with an odds ratio (OR) of 8.3, although this result was not statistically significant [28]. In contrast, another study found no significant association between smoking and CC [28]. These inconsistent outcomes suggest that further investigation is needed to clarify the relationship between smoking and CC in the Moroccan context.

**Parity:** in Morocco, parity emerges as a notable risk factor for CC, as underscored by comprehensive studies investigating the relationship between reproductive practices and CC risk. The initial investigation meticulously outlines the prevalence of pregnancies among the studied population, with an overwhelming majority having experienced at least one pregnancy. A substantial subset, encompassing 50% of cases and 30% of controls, exhibited a notable history of seven or more pregnancies, suggesting a potential connection between heightened parity and CC [25]. Subsequent research reinforces this correlation, indicating a 71.6% higher risk for women with four or more pregnancies, accompanied by an increased risk associated with an initial pregnancy occurring between ages 19 and 22 [28].

**Oral contraception:** the relationship between OC and CC risk in Morocco is illuminated by a study conducted at the INO in Rabat provides insightful data. The study, encompassing 214 cases of invasive CC and 203 controls, reports a conspicuous pattern wherein 87.5% of women diagnosed with CC had never engaged in OC use. This prevailing trend implies an absence of a straightforward association between OC usage and an augmented susceptibility to CC in this specific cohort. Intriguingly [25], among participants acknowledging OC use (80-90%), a discernible inclination towards hormonal contraception, predominantly in the form of OCs, is observed. The study unveils a notable correlation between the duration of OC use and CC risk, manifesting as a significant dose-response trend with extended periods of usage. Nonetheless, the intricacies of this relationship are accentuated by the notable 39% of participants who opted not to disclose their OC usage status [28]. Additionally, a subsequent study highlights a 60.7% elevation in CC risk associated with the utilization of OCs for six or more years (OR = 1.8) [26].

**Non-attendance for screening and underscreening:** at the INO in Rabat and the Hospital of Oncology of the CHU Ibn Rushd in Casablanca, a cross-sectional study was carried out with consecutive recruitment of 200 patients with CC from June 2008 to June 2010 [30]. This study showed a problem of late diagnosis of CC and identified factors associated with these delays. This “medical delay” was higher in rural areas, as well as among women under 50 years of age and illiterate women. In multivariate analysis, elevated risks of late-stage cancer reporting were associated with three variables: marital status, distance to the cancer center, and gynecologic bleeding [30]. The first report of the RCGC, published in 2007, recorded the incidence of all types of cancer for the year 2004 [31]. Cervical cancer ranked second (12.8% of female cancers) after breast cancer, with 235 new cases recorded. The average age of women with U CC was 53.5 years [31]. The latest edition of this registry was published in 2016, covering the period 2008-2012. According to the data from this edition [32]. Cervical cancer remains the second most common cancer in Moroccan women. It accounts for 11.2% of all female cancer cases. The highest specific incidence was noted in women over 75 years of age, with a rate of 65.2 per 100,000 women.

**Figure 3:**
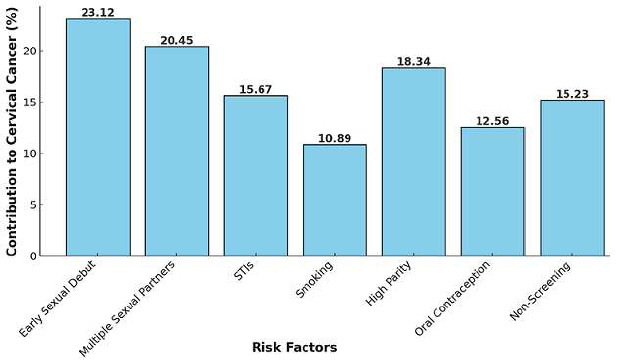
contribution of various risk factors to cervical cancer in Morocco

The Rabat cancer registry is the second Moroccan cancer registry to focus on new cancer cases diagnosed since 2005 in people living in the city of Rabat. The last report was published in 2012 and showed that CC was the second most common cancer in Moroccan women in this region (11.4% of all female cancers). The highest incidence was observed in women aged 65-74 years, with 67 CC cases per 100,000 women [33]. A third hospital-based cancer registry focuses on the population of eastern Morocco. From 2006 to 2012, this registry was housed at the Hassan II University Hospital of Fés. It was created in 2005 and reported cancer incidence data from 2006 to 2012. The CC was still in second place with 716 new cases recorded, or 9.10% of all cancers [34]. According to the IARC, approximately 3388 cases of CC were detected in Morocco in 2018, up to 2258 cases in 2012. From 1076 instances in 2012 to 2465 cases in 2018, the number of deaths has more than doubled [35]. Furthermore, the WHO/ICO 2019 study found that 13.2 million Moroccan women are at risk of contracting CC [36] (Table 3, Figure 3).

**Table 3:** key risk factors for cervical cancer in Morocco: findings and associations

Risk factor	Key findings
Early sexual debut	OR = 2.4 for intercourse <18 years
Multiple sexual partners	OR = 2.1 for ≥2 partners; conflicting results
Sexually transmitted infections	Co-infection: OR = 3.18 for cervical changes
Smoking	OR = 8.3 for heavy smoking (not significant)
High parity	71.6% higher risk for ≥4 pregnancies
Oral contraception	60.7% higher risk for use ≥6 years
Non-screening	Linked to late-stage diagnosis

### Cervical cancer prevention

**Figure 4:**
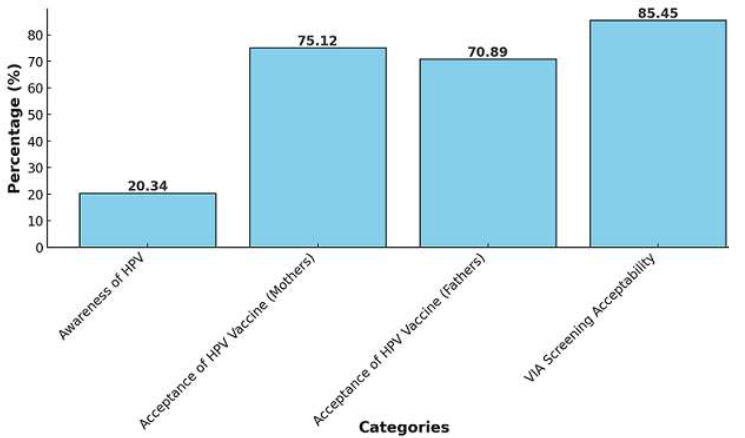
awareness and acceptability of HPV vaccination and screening in Morocco

**Primary prevention:** the introduction of HPV vaccination in Morocco marks a critical advancement in the primary prevention of CC. Since 2008, the HPV vaccine has become a crucial part of our healthcare landscape. A significant step forward occurred on October 20, 2022, upon the integration of HPV vaccination into the national schedule by the Ministry of Health in accordance with circular DP-19/2022N: 87/19, a targeted vaccination campaign has commenced. The initiative primarily focuses on administering vaccines to girls aged 11 and above, facilitated within the school premises nationwide [37]. These initiatives have been supported by the Lalla Salma Association, which has played a pivotal role in promoting vaccination coverage to reduce the prevalence of CC [38]. Research highlights significant gaps in knowledge and varying levels of acceptance regarding HPV and its vaccine among Moroccan parents. A 2012 study involving 852 parents across four regions revealed that only 4.7% were aware of HPV infection and 14.3% knew about the HPV vaccine. Acceptance rates for vaccination were low, with 32% of mothers and 45% of fathers expressing willingness to vaccinate their children, underscoring the need for targeted educational programs [39]. Similarly, a nationwide survey conducted in 2014 found slightly higher awareness levels, with 20% of participants familiar with HPV and a 27% vaccine acceptance rate. Notably, male participants showed greater acceptance (62%) compared to females (20.4%) [15]. In contrast, a more recent study focusing on parents of middle-school girls reported encouraging results, with high acceptance rates of 76.8% among mothers and 68.9% among fathers, suggesting potential for successful public health interventions [36] (Figure 4).

**Secondary prevention:** secondary prevention efforts in Morocco focus on early detection of CC through screening initiatives, though challenges remain. The Papanicolaou (Pap) smear serves as the primary screening method [40]. The National Cancer Control Plan, launched in 2010, aimed to establish structured screening programs; however, comprehensive national initiatives are yet to be fully implemented [15]. Studies highlight the prevalence of HPV even among women with normal cytological features, ranging from 15.8% in 2010 to 42.5% in 2012, emphasizing the urgency for enhanced and widespread screening efforts [15]. Screening acceptability rates are promising. A 2014 study across 24 health centers reported a 94.5% acceptance rate for Visual Inspection with Acetic Acid (VIA). Women who tested negative expressed willingness to retest every three years, while those with positive results were open to confirmatory testing [41]. The cost-effectiveness of various prevention strategies has been evaluated using a Morocco-specific Markov model. Current screening practices reduce lifetime CC risk by 14%, with a cost of $551 per year of life saved (YLS). Human papillomavirus vaccination alone, assuming 70% coverage among pre-adolescent girls, achieves a 62% risk reduction at $1,150/YLS. Combining HPV vaccination with screening offers the most substantial benefit, reducing CC risk by 69%, though at a higher cost of $2,843/YLS [42] (Table 4).

**Table 4:** cost-effectiveness of cervical cancer prevention strategies

Strategy	Lifetime cervical Cancer risk reduction	Cost (USD/YLS)
Current screening	14%	$551
Human papillomavirus vaccination	62%	$1,150
Combined strategy	69%	$2,843

**Diagnostic:** the diagnostic process for CC relies on clinical assessment, screening tests, HPV testing, imaging, and tumor staging. These modalities ensure the early detection and accurate classification of CC, which is critical for effective treatment and patient prognosis.

**Clinical examination:** for CC involves evaluating the patient´s risk factors and symptoms. Since early-stage CC often presents without noticeable signs or symptoms, regular screening is crucial for early detection. Key screening methods include.

**Pap smears:** a non-invasive test conducted during routine gynecological consultations. Cervical cells are collected for microscopic examination to identify abnormalities, often detecting precancerous changes before cancer develops.

**HPV testing:** a molecular test that identifies the presence of DNA from high-risk HPV types associated with CC. Additional complementary diagnostic procedures may include colposcopy for detailed cervical visualization, lymph node palpation, pelvic examinations, and digital rectal exams, all of which contribute to a comprehensive clinical evaluation.

**Figure 5:**
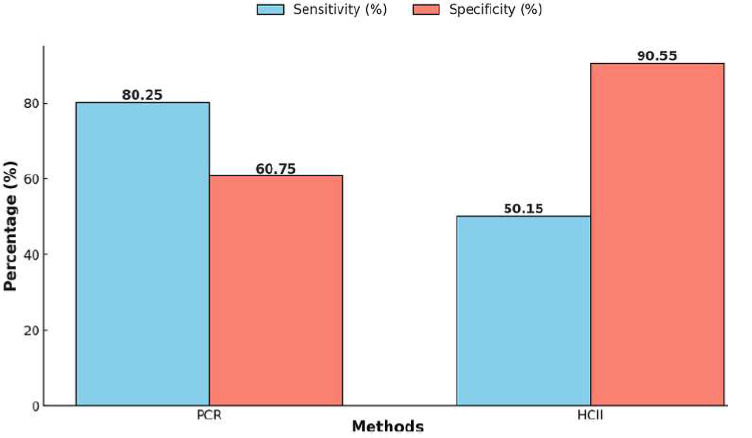
comparison of sensitivity and specificity between PCR and HCII methods

**Human papillomavirus DNA testing:** it is a critical component of CC screening, enabling the detection of high-risk HPV types. Several methods have been approved by the U.S. Food and Drug Administration (FDA) for HPV detection, including: i) Qiagen's Hybrid Capture 2 HPV DNA test (2001); ii) Hologic's Cervista HPV HR test (2009); iii) Roche's cobas 4800 HPV test (2011); iv) Gen-Probe's Aptima HPV assay (2011, acquired by Hologic in 2012); v) Becton Dickinson's BD Onclarity HPV assay (2018). In Morocco, molecular techniques such as polymerase chain reaction (PCR) are widely used in laboratories to detect HPV from cervical smears or self-collected vaginal swabs [43]. A comparative study demonstrated the strengths and limitations of PCR and hybrid capture II (HCII) methods. Polymerase chain reaction exhibited higher sensitivity (81.8%) compared to HCII (36.4%), making it more effective for identifying HPV-positive cases. However, HCII showed greater specificity (96.6%) than PCR (58.6%), reducing false-positive results [44] (Figure 5).

**Role of imaging:** imaging is a crucial tool for the classification and staging of uterine tumors, including CC. Magnetic resonance imaging (MRI) offers detailed three-dimensional visualization of the uterus, providing critical information about the lesion's size, location, and extent of involvement. This precision aids in treatment planning and prognostic evaluation. Complementary imaging techniques also play a significant role. For example, a study involving 104 CC patients undergoing pelvic computed tomography (CT) scans demonstrated that CT had high specificity (84.8%) but low sensitivity (33.3%) for detecting parametrial involvement in early-stage CC [45].

**Staging:** staging is determined clinically based on tumor size and degree of pelvic extension (Table 1). Tumor staging should be determined at the time of diagnosis and never changed. Clinical classification is preferable and more accurate in the evaluation of locally advanced disease compared with surgical staging [46,47]. Staging includes a physical examination, endoscopic procedures, and imaging modalities according to the International Federation of Gynecology and Obstetrics (FIGO) guidelines. It is not always necessary to use all modalities for every patient [48]. The FIGO system may underclassify patients. Accurate staging is crucial for treatment planning, patient counseling regarding prognosis, and verifying eligibility for research studies (Table 1) [48].

**Treatment of cervical cancer:** the optimal treatment for CC is determined by the extent of the disease and the patient´s overall health. A multidisciplinary committee, including at least one radiotherapist and one surgeon, makes the final decision on the treatment plan (Table 5) [49].

**Table 5:** staging of cervical tumors according FIGO and the AJCC

	TNM	International Federation of Gynecology and Obstetrics
Primary tumor cannot be assessed	TX	
No evidence of primary tumor	T0	
Carcinoma in situ (preinvasive)	Tis	
Cervical carcinoma confined to the cervix (without extension to uterine corpus)	T1	I
Invasive carcinoma diagnosed only by microscopy, stromal invasion with a maximum depth of 5•0 mm measured from the base of the epithelium, and horizontal spread of 7•0 mm or less; vascular space involvement, venous or lymphatic, does not affect classification	T1a	IA
Measured stromal invasion no greater than 3•0 mm and lateral spread no greater than 7•0 mm	T1a1	IA1
Measured stromal invasion greater than 3•0 mm and no greater than 5•0 mm, and horizontal spread no greater than 7•0 mm	T1a2	IA2
Clinically visible lesion confined to the cervix or microscopic lesion greater than T1a or IA2	T1b	IB
Clinically visible lesion no greater than 4•0 cm in greatest dimension	T1b1	IB1
Clinically visible lesion greater than 4•0 cm in greatest dimension	T1b2	IB2
Cervical carcinoma invades beyond the uterus but not the pelvic wall or lower third of vagina	T2	II
Tumour without parametrial invasion	T2a	IIA
Clinically visible lesion no greater than 4•0 cm in greatest dimension	T2a1	IIA1
Clinically visible lesion greater than 4•0 cm in greatest dimension	T2a2	IIA2
Tumour with parametrial invasion	T2b	IIB
Tumour extends to pelvic wall, involves lower third of vagina, causes hydronephrosis, or a combination of all symptoms, or non-functioning kidney	T3	III
Tumour involves lower third of vagina, without extending to the pelvic wall	T3a	IIIA
Tumour extends to pelvic wall, causes hydronephrosis or non-functioning kidney, or both	T3b	IIIB
Tumour invades mucosa of bladder or rectum, extends beyond the true pelvis, or both (bullous oedema is not sufficient to classify a tumour as T4 or IV)	T4	IV
Tumour invades mucosa of bladder or rectum (bullous oedema is not sufficient to classify a tumour as T4 or IV)	T4a	IVA
Tumour extends beyond the true pelvis	T4b	IVB

* TNM is a cancer staging system in which T represents the size of the primary tumor, N represents nodal involvement, and M represents metastatic disease

**Radiation therapy:** radiotherapy is a cornerstone in the treatment of CC and can be administered through external or internal methods.

**External beam radiation therapy (EBRT):** this technique delivers radiation externally in a series of sessions, typically spread over 4-5 sessions per week for approximately five weeks. External beam radiation therapy is often combined with venous chemotherapy to increase treatment efficacy and address potential microscopic disease outside the targeted radiation fields [49].

**Internal radiation therapy (Brachytherapy):** this method involves placing catheters into the uterus and vagina, typically under general or epidural anesthesia. Imaging techniques such as MRI or CT scans are used to guide the placement and delivery of the treatment. Radioactive sources, such as iridium-192, are applied to target the cervix and nearby tumor extensions selectively. When combined with EBRT, brachytherapy treatment usually spans about three days [49]. This dual approach enhances the precision and efficacy of radiotherapy, addressing both localized and broader disease involvement in CC management.

**Surgery:** the tumor and surrounding tissue are removed. In the case of very early cancers, surgery may be limited to a small area. For some more advanced tumors, the doctor will have to perform a radical hysterectomy with the complete removal of the uterus, part of the vagina, tissues adjacent to the uterus, and lymph nodes [22].

**Chemotherapy:** it is used to improve the effectiveness of radiation therapy in the treatment of CC. Chemotherapy can also be used to treat cancers that have spread to other organs (such as the lungs) [50]. For CC, chemotherapy can be combined with radiation therapy to make the treatments more effective [50]. In Morocco, the majority of CC patients are diagnosed late, needing referral for radiation, chemotherapy, or palliative treatment. A retrospective analytic study of 162 CC patients treated at the radiotherapy department of Military Hospital Mohamed V in Rabat, Morocco, between January 2005 and February 2010, revealed that more than half, 58.3% (n = 88), were treated with a combination of EBRT and concurrent cisplatin-based chemotherapy (40 mg/m^2^ weekly). Fifty-four (34%) of the patients underwent surgery as their initial treatment. Forty-six (31.4%) of these received post-operative radiotherapy or concomitant radio-chemotherapy following surgery due to positive pelvic lymph nodes, narrow or positive surgical margins, or other poor risk factors (Table 2, Table 6) [51].

**Table 6:** treatment options for cervical cancer disease at the time of diagnostic

The disease's severity at the time of diagnosis	Treatment options
The tumor is tiny and restricted to the cervix.	-Simple monitoring If the margins of the excised piece are healthy, indicating that the whole tumor was removed during ionization, and the woman desires to maintain her uterus, -If this is not the case, surgery may be necessary. It entails the removal of the uterus (hysterectomy) or the pelvic characteristics and lymph nodes in some cases. Lymph nodes in the pelvis In some circumstances, conservative surgery is recommended to preserve the uterus and is required for future pregnancy. Only the cervix (trachelectomy) and pelvic lymph nodes are removed in this case. In addition, if cancer cells infiltrate the pelvic lymph nodes, concurrent radiochemotherapy is administered
The tumor is localized to the cervix and is visible to the naked eye during a gynecological examination, but it is less than 4 cm in diameter.	-Surgery most often involves removal of the uterus, the upper third of the vagina, and the parametrium (enlarged colpo hysterectomy), as well as the ovaries and pelvic lymph nodes. -Brachytherapy is used in radiosurgery, followed by an expanded colposterectomy. -Radiotherapy combining external radiotherapy and brachytherapy can be proposed in the case of contraindication to surgery. -Concomitant radiochemotherapy is used if cancer cells are found in the pelvic lymph nodes or the surgical specimen margins.
The tumor is restricted to the cervix and is more than 4 cm in diameter, or it has spread beyond the cervix (vagina, parametrium, bladder, and rectum).	-Concurrent radiochemotherapy is the standard treatment. External radiotherapy, chemotherapy, and brachytherapy are all used in this treatment. -If radiochemotherapy is not an option, radiation therapy may be used instead -In some cases, uterine surgery is performed in conjunction with concurrent radiochemotherapy
Cancer has spread to distant organs in the form of one or more metastases.	-Chemotherapy and/or radiotherapy are used in treatment (most often external)

## Discussion

The findings from this extensive review of CC in Morocco underscore the multifaceted challenges and opportunities in understanding, preventing, diagnosing, and treating the disease within the Moroccan context. By synthesizing epidemiological data, risk factors, prevention strategies, and therapeutic approaches. Cervical Cancer remains a significant global health challenge, with its epidemiology, risk factors, and preventive measures varying widely across regions. In Morocco, CC is the second most prevalent cancer among women after breast cancer, with an incidence of 10.4 per 100,000 and a mortality rate of 5.8 per 100,000 [17]. Despite these alarming statistics, the absence of a national cancer registry and limited epidemiological studies constrain a comprehensive understanding of the disease burden. Available data suggest that CC accounts for 17.2% of the most prevalent cancers among Moroccan women, with an age-standardized incidence rate of 14.1 per 100,000 [1]. The high prevalence of HPV, the primary etiological agent of CC, further exacerbates the situation. Studies have shown HPV prevalence rates among Moroccan women with normal cervical cytology ranging from 4.2% to 42.5%, depending on study design and population characteristics [27,52,53]. These findings align with global data emphasizing the role of HPV in CC pathogenesis [22]. The global burden of CC is characterized by significant geographical disparities, with developing countries, including Morocco, bearing the brunt of the disease.

The annual incidence of CC globally was reported at 570,000 new cases in 2018, with over 85% occurring in low- and middle-income countries (LMICs) [22]. The lack of structured screening programs and limited access to healthcare in these regions, including Morocco, contribute to delayed diagnoses and poor outcomes. Retrospective studies in Morocco revealed that 85.3% of CC cases are diagnosed at intermediate or advanced stages, with stage II (43.25%) and stage III (35.6%) being the most common [18]. These findings echo trends in other LMICs, where late-stage diagnosis predominates due to barriers such as limited awareness, inadequate healthcare infrastructure, and sociocultural factors [30]. The role of HPV as a necessary but insufficient cause of CC is well established [22]. Persistent infection with high-risk oncogenic HPV subtypes, combined with cofactors such as early sexual debut, high parity, smoking, immunosuppression, and co-infections with other STIs, contributes to CC pathogenesis. Moroccan data corroborate these findings. Early initiation of sexual activity, for example, has been associated with a 2.4-fold increase in CC risk, with approximately 85% of Moroccan women reporting their first sexual experience between the ages of 14 and 19 [24,27]. Globally, early sexual debut is recognized as a significant risk factor for HPV acquisition, as demonstrated in studies from the United Kingdom, where HPV prevalence peaks in women aged 15-19, coinciding with the initiation of sexual activity [54].

Parity is another critical factor influencing CC risk. In Morocco, women with four or more pregnancies have a 71.6% higher risk of developing CC, with high parity linked to hormonal and immune changes that may promote HPV persistence [25,52,53]. This association aligns with international studies indicating that higher parity increases CC risk, likely due to repeated trauma to the cervical epithelium during childbirth, which facilitates HPV entry and integration into host cells [55-57]. OC use has also been implicated in CC risk. In Morocco, while 87.5% of women diagnosed with CC had never used OCs, a dose-response relationship was observed among users, with a 60.7% increase in CC risk associated with OC use for six or more years [26]. These findings are consistent with global studies highlighting a twofold increase in CC risk with long-term OC use [58]. The interplay between HPV and co-infections further compounds CC risk. In Morocco, co-infections with HPV and Chlamydia trachomatis were associated with a threefold increase in cervical abnormalities, while HIV-positive women exhibited higher susceptibility to high-risk HPV types and abnormal Pap smear results [21]. These patterns mirror global data showing that co-infections with STIs amplify the oncogenic potential of HPV, particularly in immunocompromised individuals [58]. Smoking, a known risk factor for squamous cell carcinoma of the cervix, has produced mixed findings in Morocco. While some studies suggest an elevated risk with smoking, others found no significant association [28]. However, international research consistently links smoking to CC through mechanisms such as local immunosuppression and DNA damage [59].

Prevention strategies, particularly HPV vaccination, offer a promising avenue for reducing CC incidence. In Morocco, HPV vaccination was introduced in 2008 and integrated into the national immunization schedule in 2022, targeting girls aged 11 and above [37]. Despite this progress, gaps in awareness and acceptance remain significant barriers. Early studies reported low awareness levels, with only 4.7% of parents aware of HPV and 14.3% aware of the vaccine, and low acceptance rates of 32% among mothers and 45% among fathers [39]. More recent data indicate improved acceptance, with 76.8% of mothers and 68.9% of fathers expressing willingness to vaccinate their daughters [36]. Globally, HPV vaccination has demonstrated substantial efficacy, with studies showing a 70% reduction in CC risk with pre-adolescent vaccination at 70% coverage [42]. These findings highlight the critical role of educational campaigns and policy interventions in enhancing vaccine uptake. Secondary prevention through screening is equally vital. In Morocco, the absence of a national screening program limits the effectiveness of initiatives such as the Papanicolaou (Pap) smear and Visual Inspection with Acetic Acid (VIA). While VIA has shown high acceptability (94.5%), its coverage remains low [41]. Cost-effectiveness analyses suggest that combining HPV vaccination with regular screening offers the most substantial reduction in CC risk, achieving a 69% risk reduction at a cost of $2,843 per year of life saved (YLS) [42].

These findings align with global recommendations advocating for integrated prevention strategies that combine vaccination and screening to maximize public health benefits. Diagnosis and treatment of CC in Morocco face significant challenges due to systemic constraints. Human papillomavirus DNA testing, a cornerstone of CC diagnosis, is underutilized due to limited access to molecular diagnostic tools. Comparative studies have shown that polymerase chain reaction (PCR) offers higher sensitivity for HPV detection, while HCII demonstrates greater specificity, underscoring the need for tailored diagnostic approaches [44]. Imaging modalities, such as MRI and CT, are critical for staging and treatment planning but remain limited in availability [45]. Most CC patients in Morocco are diagnosed at advanced stages, necessitating aggressive treatment modalities. Radiotherapy, often combined with chemotherapy, is the primary treatment for late-stage CC, while surgery is reserved for early-stage cases [49]. The reliance on cisplatin-based chemotherapy reflects the systemic delays in diagnosis and limited availability of advanced treatment options [51].

## Conclusion

The target of this CC update in Morocco was to take ownership of scientific health data in order to improve population preventive initiatives and health education in order to eliminate this preventable malignancy. Cervical cancer continues to have the highest death rate in North Africa. The ALSC established a major cancer prevention and control plan (NCCP) in 2010. The plan's objective is to use the VIA technique to detect CC early. Many hurdles have been met as a result of the data in the literature, including the training of healthcare staff, the supply of information, and patient awareness-raising. The adoption of a primary prevention program based on HPV vaccination is finally starting in Morocco in October 2022.

### What is known about this topic

- Cervical cancer is the second most common cancer among women in Morocco, with an annual incidence of 2165 cases and over 1199 deaths;
- High-risk human papillomavirus subtypes are the primary cause of CC, but the absence of a comprehensive national registry limits accurate epidemiological data;
- The NCCP, launched in 2010, prioritizes VIA-based early detection, but challenges in training and patient awareness persist.

### What this study adds

- The human papillomavirus vaccine, introduced into Morocco’s national vaccination program in 2022, targets 11-year-old girls and is a significant step toward prevention;
- Risk factors such as early sexual debut, number of partners, smoking, and parity require more in-depth investigation to guide interventions;
- Healthcare reforms, including compulsory medical coverage since 2020, promise to improve access to CC prevention and treatment services.
